# INSIGHTS FROM AFRICAN AMERICAN OLDER ADULTS ON BRAIN HEALTH RESEARCH ENGAGEMENT: NEED TO SEE THE NEED

**DOI:** 10.1093/geroni/igz038.1622

**Published:** 2019-11-08

**Authors:** Shani H Bardach, Markeda Yarbrough, Charlene Walker, Doris L Alfred, Eseosa Ighodaro, Gregory A Jicha

**Affiliations:** 1 University of Kentucky, Lexington, Kentucky, United States; 2 Bluegrass Community and Technical College, Lexington, Kentucky, United States; 3 Love’s Angels Early Childhood Development Center, Paris, Kentucky, United States

## Abstract

Recruitment of African American (AA) participants into clinical research trials in the area of aging and dementia is a major problem facing the field. Although AAs are at a significantly elevated risk of developing Alzheimer’s disease (AD), they are underrepresented in clinical trials and research studies. While previous research has identified a number of barriers to participation, relatively little is known about how to overcome these barriers and engage AA individuals in research. Photovoice may provide a novel approach to advance our understanding of AA perceptions in regards to barriers and strategies to increase AA engagement in brain aging research. The purpose of this project is to add to existing understanding of barriers and facilitators and identify strategies to enhance engagement. Three AA research advocates served as community facilitators to identify and guide groups of AA adults though an 8-10 session photovoice project. Group sessions involved discussions and sharing of images pertaining to various prompts in the area of brain health and research participation. Sessions were audio-taped and transcribed verbatim and photos were uploaded. Participants identified four categories of barriers to AA research participation: Mistrust, the belief that all research involves medication, avoidance and fear of acknowledging problems, and seeing the risks of research but not the need. Participants had various suggestions and approaches for ameliorating each of these barriers. This photovoice community engagement process revealed unique insights into barriers and opportunities for increasing AA engagement in brain aging research.

